# The Effect of Deficit Irrigation on the Quality Characteristics and Physiological Disorders of Pomegranate Fruits

**DOI:** 10.3390/plants14050720

**Published:** 2025-02-26

**Authors:** Rossana Porras-Jorge, José Mariano Aguilar, Carlos Baixauli, Julián Bartual, Bernardo Pascual, Nuria Pascual-Seva

**Affiliations:** 1 Departamento de Producción Vegetal, Universitat Politècnica de València, Camí de Vera, s/n, 46022 València, València, Spain; zrporras@doctor.upv.es; 2 Centro Experiencias Cajamar Paiporta, C. Cementerio Nuevo s/n, 46200 Paiporta, València, Spain; josemarianoaguilar@fundacioncajamar.com (J.M.A.); carlosbaixauli@fundacioncajamar.com (C.B.); 3 Estación Experimental Agraria de Elche (SST), CV-855, Km. 1, 03290 Elche, Alicante, Spain; bartual_jul@gva.es; 4 Centro Valenciano de Estudios Sobre el Riego, Universitat Politècnica de València, Camí de Vera, s/n, 46022 València, València, Spain; bpascual@upv.es

**Keywords:** ‘Mollar de Elche’, sustained deficit irrigation, regulated deficit irrigation, sensory characteristics of fruits, cracking

## Abstract

This study assesses the impact of two regulated deficit irrigation (RDI) and one sustained deficit irrigation (SDI) strategies on the fruit quality characteristics of pomegranate (*Punica granatum* L.) compared to a fully irrigated control in a Mediterranean climate. Field trials were conducted over two growing seasons at the Cajamar Experimental Center in Paiporta, Valencia, Spain. The SDI strategy, which achieved considerable water savings of approximately 50%, led to a reduction in yield (both total and marketable), as well as a decrease in the size and unit weight of the fruits. However, it also produced arils with higher dry matter content and aril juice with higher soluble solids content, all without altering the maturity index. Notably, the SDI approach resulted in increased non-marketable production due to a higher incidence of cracking, particularly during the exceptionally hot and dry summer of 2023. Although the maturity index remained unchanged across the irrigation strategies, the SDI yielded a greater percentage of pink-red rind on marketable fruits compared to the other strategies. This is important because ‘Mollar de Elche’ pomegranates are typically harvested based on their external colour. Thus, the SDI strategy could allow for earlier harvesting, potentially enhancing the commercial value, as earlier harvests often command higher prices, which may partially offset some of the reduction in marketable yield. Conversely, both RDI strategies achieved a slight water saving without compromising marketable yield or the quality characteristics of the fruit.

## 1. Introduction

‘Mollar de Elche’ (referred to as ‘ME’) is the predominant varietal group of pomegranates (*Punica granatum* L.) cultivated in Spain, accounting for over 60% of the land dedicated to pomegranate cultivation. This popularity stems from its excellent adaptation to the local edaphoclimatic conditions, high productivity, and favourable organoleptic and nutraceutical attributes. Globally, ‘ME’ is highly regarded for its exceptional flavour and antioxidant properties [1].

This study complements a companion article by the same authors published in the same Special Issue [2], which examines the physiological and agronomic responses of pomegranates to deficit irrigation. The current study focuses on how these irrigation strategies affect fruit quality and physiological disorders. Together, both studies aim to provide growers with effective irrigation management practices. In the companion paper, the growing interest in pomegranate cultivation, both in Spain and worldwide, is highlighted, particularly emphasising techniques such as deficit irrigation (DI). DI comprises irrigation practices where crops receive less water than optimal for their growth. This approach includes continuous or sustained deficit irrigation (SDI) and controlled or regulated deficit irrigation (RDI) [3,4]. SDI maintains a consistent water deficit throughout the entire crop cycle, thereby preventing substantial water stress during key phenological phases that could adversely impact marketable yield (MY). Conversely, RDI involves managing water deficits during specific stages when crops are less susceptible to water stress ([2] and references therein).

Fruit cracking and sunburn are important physiological disorders that can considerably reduce the marketable yield of pomegranate crops. Cracking, also referred as fruit split (hereinafter referred as cracking), which is the rupture of the rind, typically occurs during the final stages of fruit development [5] and is primarily caused by the pressure from rapidly expanding arils, which grow at a faster rate than the rind (also referred as skin or peel; hereinafter referred as rind) itself [6]. Other researchers [7] discovered that strategies applying the least irrigation water inputs led to the highest incidence of fruit cracking across all experimental seasons. Similarly, others [8] found that implementing RDI during the linear fruit growth phase increased cracking incidence in only one of the three tested growing seasons. This season was characterised by a sudden recovery in the plant’s water status, highlighting that fruit cracking is typically linked to abrupt changes in plant–water relationships. Furthermore, ref. [9] observed that when rainfall impacted previously water-stressed pomegranate plants during the critical final stages of fruit growth and ripening shortly before harvest, the increased pressure from the arils on the rind promoted cracking. This was due to the aril turgor rising significantly more than the rind turgor. Iscimen et al. [10] established that potassium levels and irrigation rates had a considerable effect on yield but also influenced the incidence of cracking and specific fruit quality parameters, such as shell thickness. Other contributing factors may include genetic predispositions, inadequate nutrient levels, sunburn on the rind, surface lesions from pathogens, and insect damage [6,11,12]. Sunburn, another physiological disorder, manifests as burns on the fruit’s rind when it is exposed to intense sunlight during extremely hot days, leading to a transpiration rate that exceeds the water uptake. Initially, this condition results in discolouration of small areas of the rind, but as it progresses, it can cause severe necrosis and cracking of the rind, ultimately rendering the underlying arils discoloured and unpalatable, thereby classifying them as non-marketable [11,12]. In the study by [8], sunburn on fruit increased with the SDI strategy and decreased with RDI during flowering, fruit set, and early fruit growth, compared to the control treatment in only one of three tested growing seasons. Additionally, windy conditions can exacerbate fruit damage. The friction between fruits and thorns may create wounds that can become suberified, making the fruit unsuitable for the fresh market. In severe cases, thorns can inflict deep wounds that serve as entry points for pathogens, leading to fruit decay. The primary diseases impacting pomegranate are mainly caused by several pathogens, including *Botrytis cinerea* Pers.:Fr., *Alternaria alternata* (Fr.) Keissl., *Coniella granati* (Sacc.) Petr. & Syd., and *Aspergillus niger* (Tiegh.) Speg. These pathogens typically infect the fruit during its developmental stages [13]. Other factors that may lead to non-marketable fruit include malformations and small size, but these are usually of minor significance due to the common practice of fruit thinning.

Irrigation is a critical production factor that influences the quality of pomegranate fruits, impacting key attributes such as soluble solids content, titratable acidity, dry matter, and firmness, all of which affect sensory qualities. The gross income generated from pomegranate crops is closely linked to both the marketable yield and the quality characteristics of the fruit. In a review [14] on the effect of water deficit on the physiology, phenology, vegetative growth, fruit growth, yield, and fruit quality of pomegranate, the author concluded that the results of one study could not simply be transferred to another area where conditions may be different given the variable results obtained with different systems and cultivars in different countries, which require conducting research under local conditions. The purpose of this study was to evaluate the quality characteristics of the fruit, alongside the incidence of physiological disorders, from seven-year-old pomegranate trees subjected to an SDI strategy and two RDI strategies in the early stages, ultimately aiming to establish an effective irrigation schedule for growers in the Valencian Region.

## 2. Results and Discussion

### 2.1. Fruit Characteristics

Yield, both total and marketable, was significantly influenced by the growing season (*p* ≤ 0.01), irrigation treatment (*p* ≤ 0.01), and their interaction (*p* ≤ 0.05; *p* ≤ 0.01; Table 1). Notably, marketable yields in 2023 (32.5 t ha^−1^) were higher than those in 2022 (23.3 t ha^−1^). When examining total yield, SDI recorded the lowest yield (35.5 t ha^−1^), with significant differences compared to the other irrigation strategies in both growing seasons (*p* ≤ 0.05). Similarly, for marketable yield, SDI presented the lowest values (on average 18.1 t ha^−1^); however, when compared to the control strategy, the difference was only statistically significant in 2023.

Both the growing season and irrigation strategy significantly influenced (*p* ≤ 0.01) the unit weight of the fruits. Notably, the lightest fruits were recorded in 2023 under the SDI strategy (456 g). This trend extended to fruit size, with the smallest fruits (as indicated by lower diameter measurements) also produced in 2023 and in SDI (96.9 cm). The year 2022 led to a lower overall fruit yield compared to 2023, averaging 153.6 fruits per tree in 2022 versus 255.5 in 2023. This reduced competition between fruits for photoassimilates in 2022, as suggested by [11], resulting in heavier fruits. When irrigation was restored in RDI, fruit growth accelerated, as fruits function as a strong sink for photosynthates [15], reaching sizes comparable to those found in the control strategy. The shape of the fruits was solely influenced by the growing season; on average, the fruits from 2022 exhibited a flatter shape (indicated by a higher shape index; 1.19) compared to those harvested in 2023 (1.15).

The weight of the rind and septal membranes (referred to here as the rind) was also affected by both the growing season and irrigation strategy (*p* ≤ 0.01). The lowest rind weights were associated with the fruits harvested in 2023 (213 g) and those grown using SDI (198 g), aligning with the trend in smaller fruit size and reduced unit weight. The weight of the arils was influenced by both the growing season (*p* ≤ 0.01) and the irrigation strategy (*p* ≤ 0.05), as well as the interaction between these factors (*p* ≤ 0.05). The fruits harvested in 2022 under the RDI1 strategy had the highest aril weight (360 g), while there were no significant weight differences among the irrigation strategies in 2023. The rind fraction (representing the percentage of the fruit accounted for by the rind) was only influenced by the irrigation strategy (*p* ≤ 0.05; Table 2). Fruits produced under the SDI strategy had, on average, an 11% lower rind fraction compared to those from the control strategy, indicating a 9% higher aril fraction and, consequently, a greater proportion of edible fruit. Fruits harvested in 2022 exhibited a thicker rind (4.5 mm) compared to those from 2023 (3.6 mm), which was anticipated given that 2023’s fruits were both smaller and lighter (Table 1). This observation aligns with the findings of [16], who also reported variations in rind thickness across different growing seasons. Furthermore, among the fruits collected in 2023, those produced under SDI exhibited a thinner rind (3.25 mm) compared to those from the RDI1 (3.95 mm) strategy. The dry matter content of the fruit rind remained constant, unaffected by the growing season or irrigation strategy employed.

In 2023, the percentage of pink-red rind surface on marketable pomegranates was significantly higher than in 2022 (29.8% in 2023 and 24.6% in 2022; *p* ≤ 0.01) and greater in the SDI strategy (35.3%) compared to the other irrigation strategies (22.0%, 25.6%, and 25.8 in control, RDI1, and RDI2, respectively; *p* ≤ 0.01). The colour of pomegranate rind is primarily attributed to anthocyanins, which are a class of flavonoid pigments [17]. Generally, a deeper red tone corresponds to a higher content of these pigments. As noted by [15], the colouration of pomegranate rind—a key commercial quality indicator—depends on both the concentration and type of anthocyanins present. Several factors can influence the levels of secondary metabolites, including anthocyanins, which may fluctuate seasonally. These factors include temperature, water availability, ultraviolet radiation, nutrient levels, atmospheric pollution, mechanical stress, and pathogen attack [18]. They can vary from season to season, consequently causing a variation in the colour of the pomegranate rind, both on the surface of the coloured area and in the colour of the rind itself.

The percentage of the pink-red rind area observed in pomegranates harvested in 2023 exceeded that of the previous year (*p* ≤ 0.01; Table 2). This year’s fruits exhibited a higher L* value (62.1) compared to those from 2022 (60.0), indicating a darker pigmentation, as lower luminosity values signify greater light absorption. However, neither the growing season nor the irrigation treatment appeared to significantly influence C* or h* parameters. The increased proportion of pink-red rind in SDI fruits can likely be attributed to moderate water stress experienced by the plants under this irrigation strategy. This stress could directly, as suggested by [18], or indirectly alter secondary metabolism by reducing vegetative growth, leading to the enhanced exposure of the fruits to sunlight. According to [19], this increased exposure can result in a redder appearance. These findings align with research from [20], who noted that moderate water deficits during the growth of ‘ME’ fruit only significantly affected (*p* ≤ 0.05) the h* value in the first harvest, without impacting rind colour in subsequent harvests. Additionally, [15] discovered that both SDI and, to a lesser extent, RDI fruits, exhibited lower L* values than control fruits, resulting in a darker and redder appearance. Similarly, [19] found statistical differences in L* values between growing seasons, but no significant variation between irrigation treatments in ‘ME’. Rind colouration is a critical commercial quality attribute since pomegranates of the ‘ME’ varietal group are typically harvested based on their external appearance. Thus, the increased percentage of pink-red surface on SDI fruits could allow for earlier harvests, thereby enhancing their commercial value, as earlier harvesting tends to command higher prices.

The aril fraction, which represents the weight ratio of arils to fruit, was significantly influenced by the irrigation strategy (*p* ≤ 0.01; Table 3). As anticipated, the fruits from the SDI treatment exhibited a higher aril fraction (60.8%) compared to those from the control strategy (56.1%), aligning with the findings regarding the rind-to-fruit ratio (Table 2).

Both the growing season and the irrigation strategy had a significant impact (*p* ≤ 0.01) on aril dry matter content (ADM); the fruit harvested in 2022 showed higher ADM (17.1%) compared to that harvested in 2023 (16.1%). Furthermore, the fruits from the SDI treatment presented greater ADM (17.4%) than those from the control (16.6%) and RDI1 strategies (16.5%), which were, in turn, superior to the RDI2 (16.0%) fruits.

The juice-to-fruit ratio (expressed as a percentage based on weight) was also significantly influenced by the growing season (*p* ≤ 0.01), with higher values recorded in 2022 (29.0%) compared to 2023 (22.7%). In contrast, the irrigation strategy did not exert a significant effect on this ratio, likely due to the varying responses of different strategies (particularly control and SDI) across the two growing seasons. This finding is consistent with the results reported by [15], who also found no significant effect of irrigation treatments on juice yield percentage.

The colour of the arils in the fruits was found to be influenced solely by the growing season, which affected all measured parameters. Specifically, the arils from 2023 exhibited a lighter colour (lower L*; 33.3; *p* ≤ 0.01), were more vibrant (higher C*; 36.8; *p* ≤ 0.01), and appeared redder (lower h*; 5.4; *p*≤ 0.05) compared to those from 2022 (34.0, 33.4, and 11.4 for L*, C*, and h*, respectively). These findings align with the research by [19], which similarly attributed the colour variation in the arils of ‘ME’ fruits to the growing season. Ref. [20] reported that a moderate water deficit during fruit development influenced the hue (h*), but primarily in the first harvest. They suggested that the shift towards a more intense red colour in the arils correlates with an increase in total anthocyanin content. Additionally, [21] identified six anthocyanins contributing to the red colouration of the aril juice, specifically delphinidin 3-glucoside and 3,5-diglucoside, cyanidin 3-glucoside and 3,5-diglucoside, and pelargonidin 3-glucoside and 3,5-diglucoside. When discussing the relationship between water stress and secondary metabolite production, [20] and the references therein noted that establishing a linear correlation was not possible. Mild osmotic stress from minor water limitations typically leads to reduced plant growth (as observed in the current study) while increasing the concentration of non-nitrogenous secondary metabolites. Conversely, heightened water stress triggers stomatal regulation, reducing CO_2_ assimilation due to diminished stomatal conductance, which was also evidenced in the present research. In these conditions, carbon allocation is primarily directed towards synthesising primary metabolites, thereby limiting the production of carbon-based secondary metabolites. Furthermore, the irrigation strategy played an important role in determining the rind colouration (specifically the extent of the pink-red area) of the fruits and the colour of the arils, as reported by [15]. In their studies, the SDI enhanced the coloured surface of the rind but did not significantly alter the colour of the arils. Abiotic stress from moderate water deficiency may indeed boost anthocyanin synthesis, a phenomenon previously observed in grapevines. This direct effect of water stress on the anthocyanin biosynthesis pathway has been documented by [22] and might also influence specific timing aspects of the ripening process [23]. According to [15], the timing of water stress during the growing season is crucial for the accumulation of anthocyanins in both rind and aril tissues.

Both the growing season and irrigation strategy significantly influenced the soluble solid content (SSC) of pomegranate aril juice (*p* ≤ 0.01, Table 4). Fruits harvested in 2022 exhibited a higher SSC (15.9° Brix, on average) compared to those harvested in 2023 (15.3° Brix, on average), with the SDI fruits achieving the highest SSC values (16.5° Brix, on average). This phenomenon can be attributed to a decrease in water accumulation in the SDI arils, which showed greater dry matter content (Table 3) without a corresponding increase in accumulated sugars. This observation aligns with findings by [24] in processing tomatoes and [25] in processing cherry tomatoes. The observed difference in SSC, averaging 1.25° Brix above the control arils, is remarkable, as a 1° Brix increase is considered a meaningful enhancement in fruit flavour perception [26]. According to various standards for pomegranate fruit quality [19], SSC values exceeding 14° Brix are classified as very good for sweetness. Thus, all irrigation strategies employed in this research yielded fruits with SSC values surpassing this threshold, indicating high quality. The predominant sugars in ‘ME’ pomegranate juice are fructose and glucose (reducing sugars), while the concentration of sucrose (a non-reducing sugar) generally remains low [19,27].

SSC yield was influenced by both the growing season (*p* ≤ 0.01) and irrigation strategy (*p* ≤ 0.01), as well as their interaction (*p* ≤ 0.05). On average, SSC yield in 2023 (1779 kg ha^−1^) was higher than in 2022 (1544 kg ha^−1^), while the SDI strategy yielded the lowest SSC values (1371 kg ha^−1^), consistent with expectations. The interaction analysis revealed that the lower SSC yield associated with SDI in 2023 (1343 kg ha^−1^) can be attributed to diminished overall yield, which was 26% lower than that of the control strategy. The SDI strategy not only resulted in the lowest SSC yield but also contributed to reduced plant size, consequently leading to lower soluble solid accumulation, which is related to decreased stomatal conductance [2].

Titratable acidity remained unaffected by the growing season or irrigation strategy, likely due to a high residual percentage of the sum of squares (85.3%), indicating substantial variability. As noted by [28], SSC is a poor predictor of the sweetness intensity perceived by consumers. Conversely, measuring acidity provides a more reliable estimate of acidity intensity. The acidity present in fruits and juices can obscure the relationship between SSC and sweetness perception. Consequently, the perception of sweetness is contingent on the acidity of the aril juice, making it essential to interpret SSC in conjunction with acidity, specifically through the maturity index (MI). When SSC and titratable acidity values are considered, the MI ratio compensates, revealing similar values across strategies. The MI is influenced solely by the growing season (*p* ≤ 0.05), with the highest MI corresponding to the fruits from 2022 (47.2), although in both seasons the same criterion was followed (SSC ≥ 14° Brix) and the harvest dates were practically identical.

Mellisho et al. [20] found that in the ‘ME’ cultivar, a moderate water deficit during fruit growth increased the SSC, but this effect was noted only in the first harvest and did not significantly impact titratable acidity. They also observed that the harvest date significantly influenced TA, with lower values recorded for the first harvest. Similarly, [15] reported higher SSC in ‘ME’ fruit arils using the SDI strategy; however, variations in titratable acidity resulted in notable differences in maturity index, with the highest values associated with fruits from the SDI treatment. Other authors [29] found that the effects of irrigation treatment on fruit weight, total soluble solids, total acidity, and pH of the aril juice were not significant. More recently, [30] assayed two sustained deficit irrigation treatments applying 70% and 50% ETc and found different results depending on the genotype. In the case of ‘Sefi’, differences were found with respect to the control treatment in SSC and pH (with the lowest values corresponding to the lowest water supply), while in ‘Wonderful’ no significant differences were found in these parameters; water restriction did not affect the titratable acidity in either cultivar tested. In our current study, the titratable acidity of ‘ME49’ fruit arils was comparable to the values reported for ‘ME’ by [19] and was higher than those reported by [15]. The SSC and titratable acidity values obtained led to maturity index values ranging from 42.4 to 50.8, which are consistent with the findings of [19] and surpass those of [15]. These values fall within the normal range considered for sweet varieties (31–98; [19]).

Table S1 presents the matrix of Pearson correlation coefficients for the main parameters analysed. A very strong correlation (r = 0.93; [31]) is observed between fruit size (diameter) and unit weight. According to [32], unit weight can be used as an indicator of size. The shape index (D/L; [11]) is more closely related to the maximum diameter (D) with a correlation of r = 0.72, compared to the length (L) with a correlation of r = 0.17, which justifies that the size is determined by the maximum diameter and not by the length [32].

Pomegranate fruit size, volume, and weight correlate with the number of arils per fruit, but not with the size of the arils [33]. Each aril corresponds to a fertilised ovule. Since pollination and fertilisation occur early in fruit development and each aril requires a fertilisation event, effective crop management, particularly irrigation strategies during this period, is crucial for achieving large fruits and high marketable yields [33]. Future studies will investigate the relationship between the number of arils obtained per fruit and different irrigation strategies.

The SSC shows a strong negative correlation with both total yield (r = −0.76) and marketable yield (r = −0.83). This means that as yield increases, the juice’s SSC in the arils decreases. Additionally, the dry matter content of the arils has a moderately poor negative correlation with both total yield (r = −0.66) and marketable yield (r = −0.65), indicating that the highest yields are produced by fruits with arils that have a higher water content.

The percentage of pink-red rind surface on commercial fruits exhibits a moderately poor negative correlation with both unit weight (r = −0.65) and diameter (r = −0.66). This suggests that larger and heavier fruits tend to have less pink/red on their rind surface. No correlation has been found between the colour parameters of the rind and the arils of the fruits, reinforcing the observations mentioned earlier.

### 2.2. Fruit Physiological Disorders

In terms of non-marketable yield, expressed as a percentage of the total yield by weight (Table 5), no significant differences (*p* ≤ 0.05) were found between growing seasons. However, both the irrigation strategy and the interaction between the two factors were significant (*p* ≤ 0.01). Notably, the SDI strategy resulted in a high percentage of non-marketable yield (48.4%) due to physiological disorders and decay. In 2023, the non-marketable yield for this strategy reached a peak of 57%, while the value for 2022 was also considerably high at 40%.

When analysing the incidence of various types of blemished fruits (Table 5), it is evident that sunburn and scratches were predominantly influenced by the growing season (*p* ≤ 0.01); deformed fruits were affected by the irrigation strategy (*p* ≤ 0.05), while small fruits were influenced by the interaction of both factors (*p* ≤ 0.05). Notably, the incidence of diseases, attributed to different pathogens, was negligible.

In the 2023 season, which experienced exceptionally high summer temperatures (with average and maximum temperatures of 26.1 °C and 46.8 °C, respectively), the incidence of sunburned fruits increased to 15%, compared to 10% in 2022, when average temperatures reached 26.5 °C and maximum temperatures were 39.6 °C. Conversely, 2022 saw a higher incidence of decay due to scratches (9.4%) compared to 2023 (3.4%), likely influenced by observed wind speeds. During August 2022, there were four significant gusts classified as category five on the Beaufort scale [34], reaching speeds of up to 35 km h^−1^. According to [11], windy conditions can cause fruits to rub against thorns, resulting in rind injuries that, although may suberify, render the fruits unsuitable for fresh consumption. These injuries can also lead to deeper wounds, making the fruits vulnerable to pathogenic agents that contribute to decay.

The highest incidence of deformed fruits (*p* ≤ 0.05) was noted in 2023 and in the control strategy (4.4% in both cases). The percentage of small fruits was influenced by the interaction between the growing season and the irrigation strategy, showing only significant differences between seasons under the SDI strategy.

Cracking incidence was affected by both the growing season (*p* ≤ 0.05) and the irrigation strategy (*p* ≤ 0.01), along with their interaction (*p* ≤ 0.01). Notably, the SDI strategy in 2023 exhibited a cracking incidence of 25.1%, exceeding rates from the other strategies during the same year by over eight times. In contrast, the control strategy recorded the highest cracking incidence in 2022 (8.5%) but the lowest in 2023 (1.7%).

**Figure 1:**
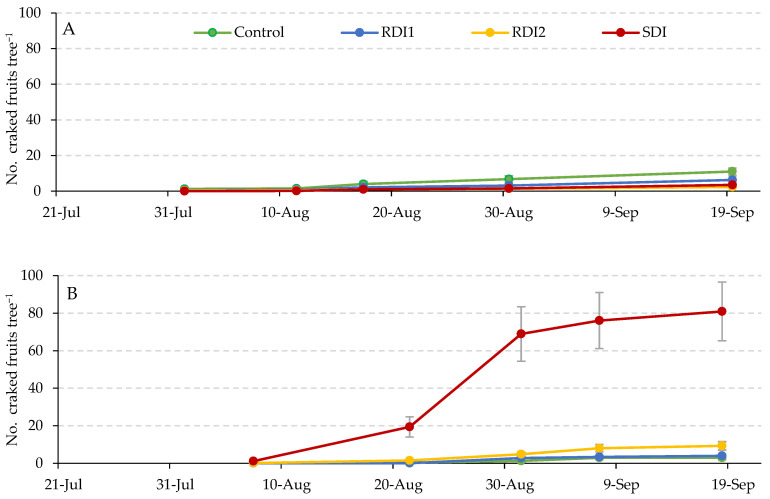
Evolution of the incidence of cracking in the four irrigation strategies tested in the two years of experimentation, 2022 (**A**) and 2023 (**B**). Vertical bars represent the standard error; their absence indicates that the bar size was less than that of the symbol used. SDI: sustained deficit irrigation; RDI1: regulated deficit irrigation (appearance of the lower buds—young fruits); RDI2: regulated deficit irrigation (open flower—fruit growth).

Figure 1 illustrates the evolution of cracking incidence across the four irrigation strategies evaluated over the two experimental years.

**Figure 2:**
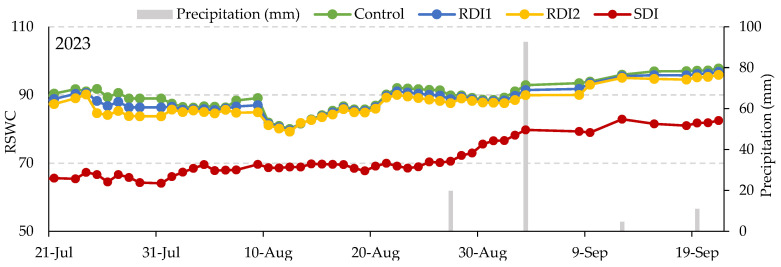
Relative soil water content [RVSWC; volumetric soil water content/volumetric soil water content at field capacity (%) at a 0.25 m depth for the four irrigation strategies tested before each irrigation event], and daily rainfall (vertical bars) from 21 July to 21 September in 2023. SDI: sustained deficit irrigation; RDI1: regulated deficit irrigation (appearance of the lower buds—young fruits); RDI2: regulated deficit irrigation (open flower—fruit growth).

Figure 2 presents the evolution of relative soil water content (volumetric soil water content/volumetric soil water content at field capacity at a depth of 0.25 m; %) for the four tested irrigation strategies, showcasing daily rainfall from 21 July to 21 September in 2023. The most significant incidences of cracking were observed with the SDI strategy in 2023, particularly during sampling on 21 and 31 August. During this period, temperatures peaked at 41.4 °C on 24 July and reached 46.8 °C on 10 August, with an average temperature of 26.9 °C from 10 August to 31 August. An increase in relative volumetric soil water content in early and late August (Figure 2) likely stemmed from irrigation management strategies defined in relation to the control strategy, as described in the Material and Methods section.

The primary cause of pomegranate cracking is fluctuations in soil moisture. This phenomenon typically arises from discrepancies in growth rates between the fruit’s rind and its arils, which is influenced by the pressure from the rapid expansion of the arils against the stretched rind [6]. The imbalance in pressure can result from a high value in aril pressure or a low value in rind resistance. Galindo et al. [9] noted that as fruit development progresses toward maturation, pomegranates become particularly vulnerable to water stress. During significant water shortages, while leaf turgor may be maintained, the fruit’s turgor can decline, leading to inhibited expansion. When rainfall or irrigation occurs after a period of water stress, the arils experience a disproportionate increase in turgor pressure compared to the rind. This disparity can cause an increase in pressure on the rind, making it more prone to cracking. These authors found strong first-order correlations between fruit water potential (Ψfruit) and stem water potential (Ψstem), indicating that Ψstem is a reliable predictor for Ψfruit. They determined that rind turgor loss and aril turgor loss occurred at approximately 1.95 and 2.46 MPa, respectively. In our 2023 experiment [2], the Ψstem did not fall below −1.125 MPa in the control strategy and −1.67 MPa in the SDI strategy. Although we did not observe a total loss of turgor in the fruit, it appears that the intensity of turgor loss may have been greater in the SDI fruits compared to those in other irrigation strategies. Similarly, in the 2022 experiment, Ψstem remained above −1.63 MPa across all strategies, indicating that fruit turgor loss was not achieved during that season either. Other authors [10] found that the number of uncracked fruits per tree increased with increasing amounts of irrigation water applied. Fruit obtained with SDI strategies that involved insufficient irrigation input showed a significant thinning of the rind, which caused the fruits to crack. They recommended that available soil water in the root zone should not fall below 50% to avoid poor fruit size and shape and increase the yield of ‘Hicaznar’ pomegranate.

The mechanical properties of fruit rind play a crucial role in sustaining internal pressure and resisting fruit cracking. According to [9], water stress can significantly alter these properties, leading to decreased extensibility and increased stiffness. Environmental factors, including temperature and relative humidity, also impact these mechanical properties. Matas et al. [35] discovered that the mechanical traits of the tomato fruit’s cuticular membrane are influenced by temperature and humidity; as temperatures rise, both stiffness and strength tend to decrease. In the summer of 2023, notably high temperatures were recorded, with an average of 26.9 °C in August and a peak of 46.8 °C on 10 August. In comparison, the maximum temperature in 2022 was considerably lower at 39.6 °C (on 15 August). The elevated temperatures seen in 2023 likely affected the cuticle, diminishing its stiffness and strength, thus increasing its vulnerability to cracking. Other environmental conditions, such as high evapotranspiration, low humidity, and drastic temperature fluctuations during the day, may also contribute to higher cracking rates [36]. The summer of 2023 experienced high evapotranspiration levels (190 mm), low average relative humidity (66%), and sharp daily temperature variations (up to 22.2 °C), which are likely linked to the increased incidence of cracking observed during fruit growth in August. As already mentioned, [35] explored how environmental conditions affect the mechanical properties of the tomato’s cuticular membrane. They found that immersing the cuticular membrane in water reduced its stiffness, relating their findings to key environmental factors that contribute to cracking: moisture on the fruit’s rind from rain or condensation coupled with high temperatures. In our 2023 study, we noted not only the high temperatures but also four rainfall events during fruit growth and ripening: 19.8 mm on 27 August, 92.5 mm on 3 September, followed by 4.7 mm on 12 September, and 11 mm on 19 September.

Genetic factors can also significantly influence fruit cracking. Different pomegranate cultivars exhibit varying levels of sensitivity to cracking [11,14,36,37,38]. Crack-resistant varieties tend to show greater water use efficiency compared to those that are more susceptible. In the current study, apart from the SDI strategy in 2023, the observed cracking levels (1.7% to 8.5% of total yield) fell below the threshold generally considered normal in Spain for the ‘ME’ varietal group of pomegranates, which is prone to cracking (5–20%; [38]).

Moreover, both fruit volume and shape—flat versus spheroidal—have been linked to susceptibility to cracking. Larger fruits, which contain more arils and exhibit a shape index greater than 1, demonstrate increased sensitivity to cracking [39]. The pressure exerted by the arils on the rind escalates with the fruit’s radius, exposing the rind of larger fruits to greater stress, thus heightening their risk of cracking. However, our study did not find a significant correlation between the incidence of cracking and parameters such as fruit weight, size (diameter and length), or shape index. According to [39], the reduction in rind elasticity seems more critical to cracking incidence than fruit volume.

In the literature, relationships have also been noted between pomegranate rind traits—such as rind thickness and the proportion of rind to aril (on a weight basis)—and cracking incidence, but these findings are not consistently observed. Mellisho et al. [20] suggested that a decreased rind fraction may correspond with higher cracking rates, while [39] found no correlation between rind thickness and cracking susceptibility. In the present study, no significant relationship was established between the incidence of cracking and either rind thickness or rind fraction; this is despite the fact that, in 2023, the SDI fruits exhibited the lowest rind thickness (*p* ≤ 0.05), a reduced rind fraction (which lacked statistical significance due to high variability between samples), and they also showed the highest incidence of cracking (*p* ≤ 0.01). It is noteworthy that the variables measured (size, unit weight, shape index, rind thickness, and rind fraction) did not demonstrate a significant correlation with cracking.

Another factor linked to fruit cracking is nutrient deficiency [5,14,36], particularly involving calcium (Ca), potassium (K), and magnesium (Mg). These nutrients are crucial as they influence the biomechanical behaviour of the fruit rind. For effective cell expansion, the accumulation of K, Mg, and Ca in the vacuole is essential for maintaining osmotic potential [40]. In litchi [41] and citrus fruits [42], low concentrations of Ca in the pericarp have been shown to contribute to fruit cracking. However, [43] reported that excessive Ca levels in pomegranate fruits could actually increase the rate of cracking. Yılmaz and Özgüven [44] discovered that cracked fruit rinds exhibited lower levels of K, as well as reduced K/Ca and K/(Ca + Mg) ratios. Cybulska et al. [45] indicated that high concentrations of Ca (greater than 2%) alter the biomechanical properties of plant tissues, making them harder and more brittle. The effect of Ca can be attributed to its ability to form bridges between pectin chains [39], whereas K imparts elasticity to the cell wall [5,11], because part of the Mg is bound to pectate in the middle lamella of cell walls, contributing to their overall structure [40].

Recently, [10] found that K application improved rind thickness of uncracked fruit and decreased fruit cracking. Chater and Garner [5] found that single foliar applications of zinc (Zn), Mg, and K in pomegranate trees reduced the incidence of fruit cracking. However, their results suggest that the physiological or biomechanical effects of these nutrient applications on the rind’s ability to withstand internal pressure were limited. They also observed a negative correlation between fruit cracking incidence and leaf concentrations of copper (Cu) and boron (B) but did not identify any other statistically significant relationships between fruit cracking and leaf nutrient concentrations. In the current study (Table 6), foliar analyses showed no significant differences in nutrient content among the various irrigation strategies, with all levels falling within the normal ranges established for ‘ME’ pomegranate [46]. Moreover, no statistically significant relationship was found between the incidence of fruit cracking and the concentrations of the analysed leaf nutrients. Given that fruit cracking is primarily caused by pressure on the rind due to the expansion of the arils within each fruit, it is important to note the considerable variability in cracking incidence both among different trees and among individual fruits on the same tree. Future research could benefit from a deeper analysis of nutrient content in fruits, particularly considering the reduced translocation of Ca within the plant.

Fruit cracking has been associated with the expression of the expansin gene [36]. During plant growth, cells produce proteins known as expansins, which play a critical role in relaxing cell wall tension and enabling turgor-driven cell enlargement. Expansins operate through a non-enzymatic mechanism by disrupting the hydrogen bonds that link cellulose fibrils and other polysaccharides [47], ultimately resulting in fruit cracking [36]. Investigating the relationship between expansin gene expression and deficit irrigation in pomegranate trees would be particularly insightful.

In summary, environmental factors recorded in 2023, such as high temperatures, elevated evapotranspiration, low humidity, significant day–night temperature fluctuations, and rainfall during the growth and ripening phases, uniformly affected all four irrigation strategies. These conditions likely impacted the cuticle, decreasing its stiffness and strength and thereby increasing susceptibility to cracking. Although the total loss of fruit turgor did not occur, there was a more pronounced decrease in turgor among fruits subjected to SDI compared to those under the other strategies. This likely resulted in an uneven increase in fruit turgor pressure; upon turgor recovery, aril turgor would have risen more significantly than that of the rind. The higher aril pressure might have rendered the rind more vulnerable to cracking, a process further exacerbated by increased soil water content, culminating in a higher incidence of fruit cracking.

## 3. Materials and Methods

### 3.1. Experimental Site and Plant Material

The field studies were conducted at the Cajamar Experimental Centre in Paiporta, Valencia, Spain (39.4175 N, 0.4184 W) over two consecutive growing seasons in 2022 and 2023. The soil at the site was uniform in the root zone depth (50 cm), with a silt loam texture, and was very slightly alkaline, slightly saline, and highly fertile (organic matter = 1.4%). Irrigation water was pumped from a well, which averaged EC 1.65 dS m^−1^. The climate is subtropical Mediterranean with hot and dry summers. Detailed information regarding soil, irrigation water, climate characteristics, and agricultural practices is available in the companion paper.

The pomegranate trees used in the experiment belonged to the ‘ME49’ clone, a selected clone of the ‘Mollar de Elche’ (‘ME’) varietal group (*Punica granatum* L.). This cultivar, part of the pomegranate gene bank at the Agricultural Experiment Station of Elche, Spain [48], is recognised for its mid-season harvest, high vigour, robust productivity, and minimal thorns. The fruit produced is large and sweet, noted for its pleasant flavour and soft seeds. Additionally, it features a pink-red rind covering a significant portion of its surface, placing it among the highly recommended varieties [11].

### 3.2. Irrigation Strategies

In the control strategy, irrigation was conducted based on practices commonly adopted by local growers, focusing on meeting crop water needs while ensuring that soil moisture reached a depth of 50 cm, without recorded water loss at that depth. Trees subjected to SDI received 50% of the water allocated to control trees. For the RDI strategies, trees were irrigated with 33.3% of their crop water needs during specific restriction periods (from BBCH 51 to BBCH 71 in RDI1 and from BBCH 61 to BBCH 73 in RDI2; Tables S2 and S3).

Volumetric soil water content (VSWC; m^3^ m^−3^) was monitored continuously using ECH_2_O EC-5 capacitance sensors linked to an Em50 data logger and managed through the ECH_2_O Utility software version 1.8.5 (Decagon Devices, Inc., Pullman, WA, USA). One sensor per replicate was placed 0.25 m beneath an emitter, as the highest density of pomegranate tree roots is found at this depth. Additional sensors placed at a depth of 0.50 m enabled the verification that moisture penetrated this deep, and that deep percolation was controlled. VSWC readings were recorded every 15 min, and variations in VSWC were utilised to assess in situ field capacity. VSWC data were standardised as a percentage of field capacity to minimise the effects of sensor calibration discrepancies. Irrigation events commenced when the VSWC in the control fell to 90% of field capacity, triggering the appropriate irrigation dose for each treatment.

### 3.3. Tree and Fruit Assessments

Leaf nutrient concentration analyses were conducted following expert recommendations [38,46]. Eighty fully expanded leaves were collected on 15 July of each year per treatment on branches free of developing fruit, with samples taken from four trees per replication block. Leaf analysis was performed at the “Las Palmerillas” laboratory in Cajamar, adhering to official methodologies [49].

Pomegranate fruits were harvested manually during two commercial pickings once they reached maturity. The fruits were deemed ready for harvest when they exhibited the characteristic colour of their variety, without any green parts, and had a SSC exceeding 14° Brix; additionally, the fruits were considered marketable only when their unit weight exceeded 220 g. The harvest dates were 13 October and 3 November 2022, as well as 16 and 31 October 2023. In this moment, 48 large, representative marketable fruits were selected for each treatment (4 fruits per tree, four trees per block, encompassing three replication blocks) and transported to the Agronomy Laboratory at the Universitat Politècnica de València for further analysis. Fruit metrics, including equatorial diameter (D) and length from calyx to base (L), were measured using a digital calliper (Powerfix Profi+, Ovibell GmbH & Co. KG, Mülheim an der Ruhr, Germany). Fruit weight (FW) was determined using a precision balance (Mettler Toledo AB204–S, Mettler-Toledo S.A.E., Cornellà del Llobregat, Spain). According to the Economic Commission for Europe [32], fruit size is assessed based on either the maximum diameter of the equatorial section or the individual unit weight, both of which were evaluated in this study.

The fruits were then peeled, and the arils were manually separated from the rind; rind thickness was measured with the digital calliper, while the arils were weighed and subsequently homogenised. Half of the arils were blended (Taurus Liquafruit Pro compact, 600 W; Taurus, Oliana, Spain) to extract the juice, following the methods described by [50]. The other half of the arils and the complete rind of each fruit were weighed separately using an analytical balance and dried at 65 °C in a forced air oven (Selecta 297; Barcelona, Spain) until reaching a constant weight, allowing for the calculation of dry weights and dry matter content.

The visual estimation of the percentage of pink-red rind surface on the selected fruits was conducted by a panel of three experts. To quantify the colour attributes of the pink-red zones, colour coordinates (L*, a*, and b*) were measured using a Minolta CR-300 chroma meter (Konica Minolta Sensing Inc., Tokyo, Japan). Measurements were taken in triplicate at three equidistant points from the equatorial region of each fruit. Similarly, the colours of the arils were assessed at four locations along the longitudinal section of the fruit. The L* value indicates lightness, with a scale ranging from 0 to 100. Chroma (C*) and hue angle (h*) were calculated using the equations C = √(a*² + b*²) [51] and h* = arctan (b*/a*) [52], respectively.

The soluble solids content (SSC, °Brix) of the juice was measured with an Atago digital refractometer (model N-20; Atago, Bellevue, WA, USA) at a laboratory temperature of 20 °C. The SSC yield (kg ha^−1^) was derived by multiplying the SSC by the total yield. Titratable acidity (TA) was determined using an acid-base potentiometer (877 Titrino plus; Metrohm Ion Analysis, Herisau, Switzerland) with 0.1 N NaOH to titrate the sample up to pH 8.1, reporting the results as grammes of citric acid per litre (g of citric acid L^−1^). The maturity index (MI) was calculated from these values as the SSC/TA ratio.

Cracked fruits were monitored periodically on each tree leading up to harvest, with any cracked fruits removed following each assessment. Upon harvest, a thorough visual inspection of all fruits was performed to identify physiological disorders such as sunburn, cracking, and other anomalies (including scratches, small size, and deformities). Fruits exhibiting blemishes (including previously harvested cracked fruits) were classified as non-marketable, and this was expressed as a percentage of non-marketable fruit compared to the total of marketable and non-marketable fruit.

### 3.4. Experimental Design and Statistical Analysis

The experimental layout utilised a randomised complete block design with three replicates. Each experimental plot consisted of three rows of trees, each containing six trees. The analysis was conducted on the four central trees of the middle row, selected for their consistent characteristics (height, ground shaded area, trunk cross-section, etc.) in accordance with the recommendations of [53].

The results for the various parameters were analysed using analysis of variance (ANOVA) via Statgraphics Centurion XVII [54]. Prior to analysis, percentage data were arcsin √ transformed. The least significant difference (LSD) at a 0.05 probability level was used as the criterion for mean separation. Additionally, the Pearson correlation coefficient was computed to evaluate potential interdependencies among the quantitative parameters.

## 4. Conclusions

The characteristics of fruits are influenced by both the growing season and the irrigation strategy employed. The largest fruits with the highest unit weight are produced during the season with the lowest yield. In comparison to the control strategy, SDI tends to reduce fruit size and unit weight. However, this strategy also results in arils with higher dry matter content and aril juice that possesses greater soluble solids concentration, all without impacting the maturity index. One downside of this SDI is the increase in non-marketable yields, particularly due to cracking incidents, which are more likely during extremely hot and dry summers. The RDI strategies that were tested did not compromise either commercial yield or fruit quality, and they represented a slight water saving, making them recommendable irrigation strategies for pomegranate cultivation. Given the lack of significant differences between both RDI strategies, it would be interesting to continue with this study, lengthening the restriction period between the flowering and early fruit growth phase with colour change (BBCH51-BBCH73).

## Figures and Tables

**Table 1:** Effect of the growing season and the irrigation strategy on the total (TY; t ha^−1^) and marketable yield (MY; t ha^−1^), unit weight (UW; g) with its partitioning into rind (RW) and arils (AW) weight, and size [maximum diameter of the equatorial section (D; mm)] and shape index (D/L, being L the length, mm) of the fruits.

		TY		MY		UW		D		D/L		RW		AW	
Growing season (GS)															
	2022	36.74	b	23.33	b	572.78	a	105.27	a	1.19	b	241.17	a	331.61	a
	2023	52.54	a	32.54	a	500.86	b	100.00	b	1.15	a	213.02	b	287.83	b
	LSD	2.89		2.12		22.37		1.44		0.02		19.38		12.86	
Irrigation strategy (IS)															
	Control	46.35	a	29.40	b	539.61	a	103.72	a	1.17		239.77	a	299.84	b
	RDI1	48.10	a	32.53	a	566.66	a	104.05	a	1.16		239.09	a	327.56	a
	RDI2	48.56	a	31.73	ab	540.04	a	103.20	a	1.18		231.13	a	308.91	b
	SDI	35.54	b	18.09	c	500.98	b	99.56	b	1.17		198.39	b	302.58	b
	LSD	4.08		2.99		31.64		2.04		0.02		27.41		18.19	
GS × IS															
	2022-Control	38.37	b	22.91	bc	550.30		105.68		1.20		245.53		304.77	cd
	2022-RDI1	39.36	b	25.57	b	613.44		106.27		1.16		253.89		359.55	a
	2022-RDI2	38.20	b	26.09	b	581.46		106.87		1.20		253.48		327.98	bc
	2022-SDI	31.02	c	18.75	cd	545.94		102.25		1.18		211.78		334.15	ab
	2023-Control	54.34	a	35.88	a	528.92		101.75		1.15		234.01		294.91	de
	2023-RDI1	56.83	a	39.48	a	519.87		101.83		1.16		224.30		295.58	de
	2023-RDI2	58.92	a	37.37	a	498.62		99.54		1.15		208.78		289.84	de
	2023-SDI	40.05	b	17.43	d	456.01		96.88		1.15		185.00		271.01	e
	LSD	5.77		4.23		44.75		2.89		0.03		38.77		25.72	
Source (df)		% sum of squares													
S (1)		44.04	**	23.75	**	16.22	**	19.66	**	9.19	**	3.99	**	17.92	**
IS (3)		19.96	**	37.70	**	6.88	**	9.18	**	1.42	ns	5.77	**	4.37	*
GS × IS (3)		3.22	*	10.60	**	2.72	ns	1.19	ns	2.68	ns	0.70	ns	4.59	*
Residual (88 ^Z^/184 ^Y^)		32.78		27.95		74.19		69.97		86.71		89.54		73.11	
Standard deviation		7.12		5.22		78.57		5.07		0.06		68.07		45.16	

Mean values followed by different lowercase letters in each column indicate significant differences at *p* ≤ 0.05 using the LSD test. Degrees of freedom (df): ^Z^ for TY and MY and ^Y^ for UW, D, D/L, RW, and AW. ns: no significant difference; ** (*): significant differences at *p* ≤ 0.01 (*p* ≤ 0.05).

**Table 2:** Effect of the growing season and irrigation strategy on the rind characteristics [rind fraction (RF, %), rind thickness (RT, mm), rind dry matter content (RDM, %), the pink-red zone of the rind (% with respect to the entire surface), and the CIE L*C*h* colour space parameters of the pink-red zone of the rind (L* indicates lightness, C* represents chroma, and h* is the hue angle)].

		RF		RT		RDM		Pink-Red Zone of the Rind							
								%		L*		C*		h*	
Growing season (GS)															
	2022	41.26		4.51	a	26.25		24.58	b	60.04	b	45.90		51.62	
	2023	42.28		3.61	b	24.37		29.79	a	62.07	a	45.65		49.05	
	LSD	2.26		0.22		1.93		3.47		1.46		0.88		2.66	
Irrigation strategy (IS)															
	Control	43.93	a	3.96		26.46		21.98	b	61.20		45.48		49.89	
	RDI1	41.92	ab	4.19		23.72		25.63	b	60.68		46.01		50.12	
	RDI2	42.08	ab	4.13		26.27		25.83	b	62.59		45.18		52.15	
	SDI	39.16	b	3.96		24.81		35.31	a	59.76		46.41		49.19	
	LSD	3.19		0.32		2.73		4.91		2.06		1.24		3.76	
GS × IS															
	2022-Control	43.83		4.33	ab	27.88		20.00		58.97		46.32		48.46	
	2022-RDI1	40.69		4.43	a	24.07		22.71		60.21		45.91		51.97	
	2022-RDI2	42.62		4.63	a	27.56		23.54		62.41		45.14		54.60	
	2022-SDI	37.90		4.67	a	25.50		32.08		58.58		46.21		51.45	
	2023-Control	44.03		3.60	cd	25.03		23.96		63.43		44.65		51.32	
	2023-RDI1	43.15		3.95	bc	23.36		28.54		61.14		46.11		48.27	
	2023-RDI2	41.53		3.64	cd	24.99		28.13		62.78		45.23		49.69	
	2023-SDI	40.41		3.25	d	24.12		38.54		60.95		46.62		46.93	
	LSD	4.51		0.45		3.86		6.94		2.91		1.76		5.31	
Source (df)		% sum of squares													
GS (1)		0.39	ns	41.27	**	3.83	ns	2.10	**	1.90	*	0.08	ns	0.93	ns
IS (3)		4.37	*	2.14	ns	5.47	ns	7.57	**	1.94	ns	1.18	ns	0.69	ns
GS × IS (3)		0.88	ns	5.98	*	0.81	ns	0.08	ns	1.15	ns	0.90	ns	1.42	ns
Residual (184)		94.35		50.61		89.90		90.21		95.02		97.85		96.97	
Standard deviation		8.08		0.52		4.75		17.28		7.25		4.38		13.23	

Mean values followed by different lowercase letters in each column indicate significant differences at *p* ≤ 0.05 using the LSD test. df: degrees of freedom; ns: no significant difference; ** (*): significant differences at *p* ≤ 0.01 (*p* ≤ 0.05).

**Table 3:** Effect of the growing season and irrigation strategy on the characteristics of the arils [aril fraction (AF, %), dry matter content (ADM, %), juice/fruit ratio (J/F, % based on weight), and on the CIE L*C*h* colour space parameters (L* indicates lightness. C* represents chroma and h* is the hue angle)].

		AF		ADM		J/F		L*		C*		h*	
Growing season (GS)													
	2022	58.74		17.08	a	29.03	a	33.95	a	33.37	b	11.43	a
	2023	57.72		16.13	b	22.65	b	33.31	b	36.81	a	5.38	b
	LSD	2.30		0.41		1.37		0.63		1.54		2.23	
Irrigation strategy (IS)													
	Control	56.07	b	16.57	b	25.37		33.98		35.10		10.79	
	RDI1	58.08	ab	16.51	b	27.19		33.74		36.56		7.45	
	RDI2	57.92	ab	15.96	c	24.89		33.30		33.46		6.92	
	SDI	60.84	a	17.37	a	25.92		33.51		35.24		8.45	
	LSD	3.26		0.58		1.93		0.88		2.17		3.15	
GS × IS													
	2022-Control	56.17		17.09		26.65	bc	34.36		34.60		13.39	
	2022-RDI1	59.31		17.16		30.90	a	34.01		34.03		10.91	
	2022-RDI2	57.38		16.21		27.08	b	33.23		32.60		9.17	
	2022-SDI	62.1		17.85		31.50	a	34.22		32.25		12.25	
	2023-Control	55.97		16.05		24.08	cd	33.61		35.60		8.19	
	2023-RDI1	56.85		15.86		23.47	d	33.46		39.08		4.00	
	2023-RDI2	58.47		15.71		22.70	de	33.38		34.32		4.68	
	2023-SDI	59.59		16.89		20.35	e	32.80		38.24		4.64	
	LSD	4.60		0.82		2.73		1.25		3.07		4.46	
Source (df)		% sum of squares											
GS (1)		0.39	ns	15.88	**	28.59	**	2.80	*	11.45	**	16.68	**
IS (3)		4.37	ns	17.65	**	2.07	ns	1.76	ns	4.68	ns	4.01	ns
GS × IS (3)		0.88	ns	1.45	ns	7.42	**	2.17	ns	4.36	ns	0.72	ns
Residual (184)		94.35		65.02		61.92		93.26		79.51		78.59	
Standard deviation		8.08		1.00		4.80		1.90		4.66		6.76	

Mean values followed by different lowercase letters in each column indicate significant differences at *p* ≤ 0.05 using the LSD test. df: degrees of freedom; ns: no significant difference; ** (*): significant differences at *p* ≤ 0.01 (*p* ≤ 0.05).

**Table 4:** Effect of growing season and irrigation strategies on pomegranate aril juice soluble solids content (SSC, °Brix), SSC yield (kg ha^−1^), titratable acidity (TA, g citric acid L^−1^), and maturity index (MI = SSC/TA).

		SSC		SSC Yield		TA		MI	
Growing season (GS)									
	2022	15.90	a	1544	b	0.35		47.21	a
	2023	15.28	b	1779	a	0.35		43.50	b
	LSD	0.17		124		0.02		3.15	
Irrigation strategy (IS)									
	Control	15.23	b	1657	a	0.33		46.59	
	RDI1	15.29	b	1827	a	0.35		45.83	
	RDI2	15.36	b	1792	a	0.35		44.40	
	SDI	16.48	a	1371	b	0.37		44.61	
	LSD	0.23		175		0.03		4.46	
GS × IS									
	2022-Control	15.65		1481	cd	0.31		50.82	
	2022-RDI1	15.61		1717	bc	0.36		48.35	
	2022-RDI2	15.76		1579	cd	0.37		43.90	
	2022-SDI	16.60		1398	d	0.37		45.76	
	2023-Control	14.82		1833	ab	0.35		42.36	
	2023-RDI1	14.97		1936	ab	0.35		43.31	
	2023-RDI2	14.96		2005	a	0.34		44.90	
	2023-SDI	16.36		1343	d	0.38		43.45	
	LSD	0.33		248		0.04		6.31	
Source (df)	% sum of squares								
S (1)		18.47	**	9.87	**	0.04	ns	5.47	*
IS (3)		50.02	**	22.98	**	7.76	ns	1.29	ns
GS × IS (3)		2.65	ns	6.00	*	6.90	ns	4.83	ns
Residual (184)		28.86		61.15		85.30	ns	88.4	
Standard deviation		0.41		306		0.05		7.78	

Mean values followed by different lowercase letters in each column indicate significant differences at *p* ≤ 0.05 using the LSD test. df: degrees of freedom; ns: no significant difference; ** (*): significant differences at *p* ≤ 0.01 (*p* ≤ 0.05).

**Table 5:** Effect of growing season and irrigation strategy on non-marketable yield (in terms of % yield by weight) and its partitioning into sunburned, cracked, scratched, deformed, and small fruits.

		Total		Sunburned		Cracked		Scratched		Deformed		Small Fruits	
Growing season (GS)													
	2022	36.71		10.27	b	5.70	b	9.37	a	2.48	b	8.90	
	2023	39.25		15.41	a	7.99	a	3.42	b	4.40	a	8.04	
	LSD	3.01		1.54		2.05		1.19		0.84		1.39	
Irrigation strategy (IS)													
	Control	36.85	b	12.93		5.09	b	5.81		4.37	a	8.65	
	RDI 1	32.54	c	11.96		3.39	b	5.92		3.08	b	8.19	
	SDI	34.14	bc	12.38		3.24	b	6.58		3.68	ab	8.27	
	RDS	48.40	a	14.08		15.67	a	7.25		2.63	b	8.78	
	LSD	4.26		2.17		2.90		1.69		1.18		1.97	
GS × IS													
	2022-Control	40.35	b	10.99		8.53	b	8.45		3.39		9.00	ab
	2022-RDI 1	34.81	bcd	10.17		4.62	bcd	8.77		2.50		8.75	ab
	2022-RDI 2	31.57	cd	8.72		3.41	cd	10.03		2.26		7.15	b
	2022-SDI	40.12	b	11.20		6.23	bc	10.23		1.77		10.70	a
	2023-Control	33.35	cd	14.87		1.65	d	3.18		5.36		8.30	ab
	2023-RDI 1	30.27	d	13.76		2.15	cd	3.07		3.66		7.64	b
	2023-RDI 2	36.71	bc	16.04		3.06	cd	3.14		5.09		9.38	ab
	2023-SDI	56.69	a	16.97		25.11	a	4.28		3.49		6.85	b
	LSD	6.02		3.08		4.10		2.39		1.67		2.78	
Source (df)		% sum of squares											
GS (1)		1.44	ns	31.48	**	1.75	*	51.46	**	17.24	**	1.52	ns
IS (3)		34.36	**	3.02	ns	35.08	**	1.94	ns	8.05	*	0.50	ns
GS × IS (3)		19.18	**	2.72	ns	32.21	**	0.51	ns	1.68	ns	9.52	*
Residual (88)		45.02		62.78		30.96		46.10		73.03		88.46	
Standard deviation		7.42		3.79		5.05		2.94		2.06		3.43	

Mean values followed by different lowercase letters in each column indicate significant differences at *p* ≤ 0.05 using the LSD test. df: degrees of freedom; ns: no significant difference; ** (*): significant differences at *p* ≤ 0.01 (*p* ≤ 0.05).

**Table 6:** Effect of growing season and irrigation strategy on leaf macronutrient (nitrogen, phosphorus, potassium, calcium, and magnesium; %) and micronutrient (iron, copper, manganese, and zinc; mg L^−1^) concentrations in adult leaves (collected on 15 July of each year) of pomegranate trees of clone 49 ‘Mollar de Elche’. Average values of four trees per replication block.

		Macronutrients										Micronutrients							
		N		P		K		Ca		Mg		Fe		Cu		Mn		Zn	
Growing season (GS)																			
	2022	1.82	b	0.15		0.85		1.53	b	0.35		145.3	a	6.33		26.7	b	20.2	
	2023	2.01	a	0.16		0.89		1.84	a	0.41		114.2	b	8.42		40.0	a	16.2	
	LSD	0.18		0.02		0.11		0.14		0.07		24.54		2.34		4.47		4.40	
Irrigation strategy (IS)																			
	Control	2.04		0.16		0.86		1.71		0.38		124.3		8.50		34.0		19.3	
	RDI1	1.88		0.15		0.88		1.73		0.35		131.3		6.50		33.3		17.7	
	RDI2	1.92		0.15		0.91		1.62		0.38		144.8		7.50		33.3		18.5	
	SDI	1.82		0.14		0.83		1.68		0.41		118.5		7.00		32.7		17.2	
	LSD	0.25		0.02		0.16		0.20		0.10		34.71		3.31		6.32		6.22	
GS × IS																			
	2022-Control	1.99		0.16		0.79		1.56		0.33		140.0		7.33		27.3		19.7	
	2022-RDI1	1.75		0.14		0.91		1.66		0.36		149.7		5.00		27.0		21.7	
	2022-RDI2	1.75		0.13		0.89		1.37		0.37		146.0		7.00		24.3		21.0	
	2022-SDI	1.79		0.15		0.81		1.51		0.34		145.7		6.00		28.0		18.3	
	2023-Control	2.10		0.17		0.92		1.86		0.43		108.7		9.67		40.7		19.0	
	2023-RDI1	2.00		0.16		0.85		1.81		0.35		113.0		8.00		39.7		13.7	
	2023-RDI2	2.09		0.18		0.93		1.86		0.38		143.7		8.00		42.3		16.0	
	2023-SDI	1.86		0.13		0.85		1.84		0.48		91.3		8.00		37.3		16.0	
	LSD	0.36		0.03		0.22		0.28		0.14		49.09		4.69		8.93		8.79	
Source (df)		% sum of squares																	
GS (1)		19.45	*	10.38	ns	2.93	ns	52.29	**	15.19	ns	25.22	*	16.31	ns	68.58	*	16.80	ns
IS (3)		14.04	ns	11.32	ns	6.56	ns	3.81	ns	6.21	ns	10.02	ns	8.22	ns	0.34	ns	2.86	ns
GS × IS (3)		5.80	ns	28.85	ns	8.05	ns	7.66	ns	14.33	ns	9.08	ns	1.96	ns	3.69	ns	8.11	ns
Residual (88)		60.70		49.44		82.47		36.23		64.27		55.68		73.51		27.39		72.23	
Standard deviation		0.21		0.02		0.13		0.16		0.08		28.36		2.71		5.16		5.08	

Mean values followed by different lowercase letters in each column indicate significant differences at *p* ≤ 0.05 using the LSD test. df: degrees of freedom; ns: no significant difference; ** (*): significant differences at *p* ≤ 0.01 (*p* ≤ 0.05).

## Data Availability

Dataset available on request from the authors.

## Supplementary Materials

The following supporting information can be downloaded at https://www.mdpi.com/article/10.3390/plants14050720/s1: Table S1. Matrix of Pearson correlation coefficients of the parameter values corresponding to the yield [total (TY) and marketable (MY] and characteristics of the fruits [unit weight (UW) and its partitioning in arils (AW) and rind (RW), size (equatorial diameter (D) and length (L)) and shape index (SI)], rind [R; thick (RT), dry matter content (RDM), pink-red zone of the rind (% with respect to the entire surface), and CIE L*C*h* colour space parameters of the pink-red zone of the rind)], aril [A; dry matter content (ADM), CIE L*C*h* colour space parameters] and aril juice soluble solids content (SSC), titratable acidity (TA) and maturity index (MI). Table S2. Irrigation restriction periods in the regulated deficit irrigation strategies for 2022 and 2023 [2]. Table S3. Irrigation water applied corresponding to irrigation strategies (IS) assayed, expressed as a percentage of the irrigation water requirements applied [2].
