# Supplementary material for: The Effect of Deficit Irrigation on the Quality Characteristics and Physiological Disorders of Pomegranate Fruits
Source: Plants (Basel). 2025 Feb 26;14(5):720. doi: 10.3390/plants14050720 (PMC11901697; doi:10.3390/plants14050720)
Supplement: Supplementary file 1 [file plants-14-00720-s001.zip › plants-3481037-supplementary.pdf]

Table S1: Matrix of Pearson correlation coefficients of the parameter values corresponding to the yield [total (TY) and marketable (MY)] and characteristics of the fruits [unit weight (UW) and its partitioning in arils (AW) and rind (RW), size (equatorial diameter (D) and length (L)) and shape index (SI)], rind [R; thick (RT), dry matter content (RDM), pink-red zone of the rind (% with respect to the entire surface; %PR), and CIE L*C*h* colour space parameters of the pink-red zone of the rind)], aril [A; dry matter content (ADM), CIE L*C*h* colour space parameters] and aril juice soluble solids content (SSC), titratable acidity (TA) and maturity index (MI).

	TY	MY	UW	AW	RW	TR	D	L	D/L	ADM	RDM	% PR	RL*	RC*	RH*	AL*	AC*	AH*	SSC	TA	MI
**TY**	1																				
**MY**	0.91***	1																			
**UW**	-0.22	0.04	1																		
**AW**	-0.41*	-0.17	0.88***	1																	
**RW**	0.04	0.26	0.86***	0.51	1																
**TR**	-0.49*	0.19	0.59**	0.59**	0.42*	1															
**D**	-0.29	0	0.93***	0.81***	0.81***	0.61**	1														
**L**	0.01	0.25	0.91***	0.73***	0.86***	0.45*	0.8***	1													
**D/L**	-0.50*	0.31	0.48*	0.49*	1.35	0.52**	0.72***	1.17	1												
**ADM**	-0.67***	-0.66***	0.17	0.11	0.17	0.41*	0.08	0.05	0.10	1											
**RDM**	-0.29	-0.23	0.07	0.07	0.06	0.31	0.16	0.00	0.28	0.22	1										
**% PR**	-0.12	-0.27	-0.65***	-0.50*	-0.64***	-0.43*	-0.66***	-0.69***	-0.26	0.16	-0.20	1									
**RL***	0.52**	0.49*	0.01	-0.21	0.23	-0.16	-0.05	0.08	-0.20	-0.26	-0.04	-0.23	1								
**RC***	-0.29	-0.35	-0.31	-0.1	-0.45*	-0.03	-0.25	-0.37	0.02	0.06	0.01	0.29	-0.63***	1							
**RH***	-0.14	0.02	0.4*	1.27	0.43*	0.53**	1.34	0.37*	0.13	0.23	0.13	-0.38	0.58**	-0.55**	1						
**AL***	-0.09	0.04	0.28	0.14	0.36*	0.28	0.33	0.23	0.28	0.16	0.07	-0.19	-0.22	0.14	0.05	1					
**AC***	0.39*	0.28	-0.29	-0.36*	0.87	-0.37*	-0.34	-0.15	-0.40*	-0.13	-0.29	0.18	0.26	0.03	-0.09	-0.29	1				
**A H***	-0.46*	-0.27	0.29	0.29	0.22	0.50*	0.31	0.23	0.26	0.44*	0.53**	-0.13	-0.14	0.01	0.38*	0.28	-0.03	1			
**SSC**	-0.76***	-0.83***	-0.12	0.09	-0.31	0.19	-0.08	-0.29	0.25	0.58**	0.17	0.43*	-0.50*	0.47*	-0.11	0.03	-0.17	0.19	1		
**TA**	-0.10	-0.13	-0.02	-0.07	0.03	-0.07	-0.01	-0.07	0.09	0.09	-0.17	0.29	-0.02	-0.12	0.04	0.15	-0.10	-0.23	0.09	1	
**MI**	-0.19	-0.17	0.13	0.24	-0.02	0.18	0.08	0.12	-0.02	0.15	0.15	-0.23	-0.14	0.23	0.03	-0.13	0.03	0.33	0.19	-0.93***	1

Table S2. Irrigation restriction periods in the regulated deficit irrigation strategies for 2022 and 2023 [2].

Strategy	Beginning	End	2022		2023	
			From	To	From	To
RDI1	The end of stage 1 (BBCH 51)	Young fruit of second flowering (BBCH 71)	April 26	June 19	April 19	June 4
RDI2	First open flower (BBCH 61)	Fruit growth of second flowering (BBCH 73)	May 16	June 26	April 29	June 11

Table S3. Irrigation water applied corresponding to irrigation strategies (IS) assayed, expressed as a percentage of the irrigation water requirements applied [2].

IS	February	March	April	May	June	July	August	September	October
Control	100%								
RDI1	100%			33.3%		100%			
RDI2	100%			33.3%		100%			
SDI	50%								
